# Near infrared photoimmunotherapy prevents lung cancer metastases in a murine model

**DOI:** 10.18632/oncotarget.3850

**Published:** 2015-05-13

**Authors:** Kazuhide Sato, Tadanobu Nagaya, Yuko Nakamura, Toshiko Harada, Peter L. Choyke, Hisataka Kobayashi

**Affiliations:** ^1^ Molecular Imaging Program, Center for Cancer Research, National Cancer Institute, Bethesda, MD 20892-1088, USA

**Keywords:** near infrared photoimmunotherapy, metastasis prevention, targeted metastasis treatment, HER2 receptor

## Abstract

Near infrared photoimmunotherapy (NIR-PIT) is a new cancer treatment that combines the specificity of intravenously injected antibodies with the acute toxicity induced by photosensitizers after exposure to NIR-light. Herein, we evaluate the efficacy of NIR-PIT in preventing lung metastases in a mouse model. Lung is one of the most common sites for developing metastases, but it also has the deepest tissue light penetration. Thus, lung is the ideal site for treating early metastases by using a light-based strategy. *In vitro* NIR-PIT cytotoxicity was assessed with dead cell staining, luciferase activity, and a decrease in cytoplasmic GFP fluorescence in 3T3/HER2-luc-GFP cells incubated with an anti-HER2 antibody photosensitizer conjugate. Cell-specific killing was demonstrated in mixed 2D/3D cell cultures of 3T3/HER2-luc-GFP (target) and 3T3-RFP (non-target) cells. *In vivo* NIR-PIT was performed in the left lung in a mouse model of lung metastases, and the number of metastasis nodules, tumor fluorescence, and luciferase activity were all evaluated. All three evaluations demonstrated that the NIR-PIT-treated lung had significant reductions in metastatic disease (*p < 0.0001, Mann-Whitney *U*-test) and that NIR-PIT did not damage non-target tumors or normal lung tissue. Thus, NIR-PIT can specifically prevent early metastases and is a promising anti-metastatic therapy.

## INTRODUCTION

Cancer metastasis is the main cause of cancer death. After patients develop metastatic disease in lung, the treatment options become limited to palliative surgical resection of visible oligometastases or systemic chemotherapy with more advanced disease [1, 2]. Once established, metastatic foci are even more difficult to treat because they acquire their own blood supply and microenvironment [2]. Thus, a therapy that could treat early lung metastases with minimal damage to the surrounding normal organs would be welcome.

The concept of targeted light therapy with photosensitizers was established over three decades ago [3, 4]. However, most photosensitizers do not target tumors with sufficient selectivity resulting in off-target side effects. Attempts to improve photosensitizers have been limited by the hydrophobicity of traditional photosensitizers used in photodynamic therapy (PDT), which dominate the pharmacokinetics of antibody-PDT conjugates. However, if the photosensitizer is highly hydrophilic, then the pharmacokinetics of an antibody-photosensitizer conjugate are governed by the antibody, and true targeting is possible. IRDye700DX (IR700, a silica-phthalocyanine dye) is a highly hydrophilic photosensitizer that is excited by near infrared (NIR) light at a wavelength of 690 nm. If it is conjugated to a monoclonal antibody, this antibody photosensitizer conjugate (APC) is able to treat tumors, a process that has been called “near infrared photoimmunotherapy” (NIR-PIT). This treatment differs from traditional PDT not only in the hydrophilicity of the photosensitizer but also in its reliance on NIR-light that has better tissue penetration than lower wavelength light used with PDT. NIR-PIT APCs demonstrate similar intravenous pharmacokinetics to naked antibodies, which results in highly targeted accumulation with minimal non-target binding. If they are bound to targeted cells and exposed to 690 nm NIR-light, these APCs induce rapid, selective and irreversible damage to the cell membrane. *In vitro* studies have demonstrated that NIR-PIT is highly target cell-specific; therefore, non-target expressing cells suffer no toxic effects, even if they are immediately adjacent [5]. Cell membrane rupture can be demonstrated within minutes of exposure to NIR-light in targeted cells [6, 7]. Because of its high selectivity and minimal side effects as well as the excellent transmission of NIR-light in lung tissue, NIR-PIT is a potential therapy to prevent further growth of early lung metastases. Here, we investigate the efficacy of NIR-PIT in a murine model of early-stage lung metastases.

## RESULTS

### Characterization of cell line and target-specific cell killing with NIR-PIT in *in vitro* 2D culture

**Figure 1:**
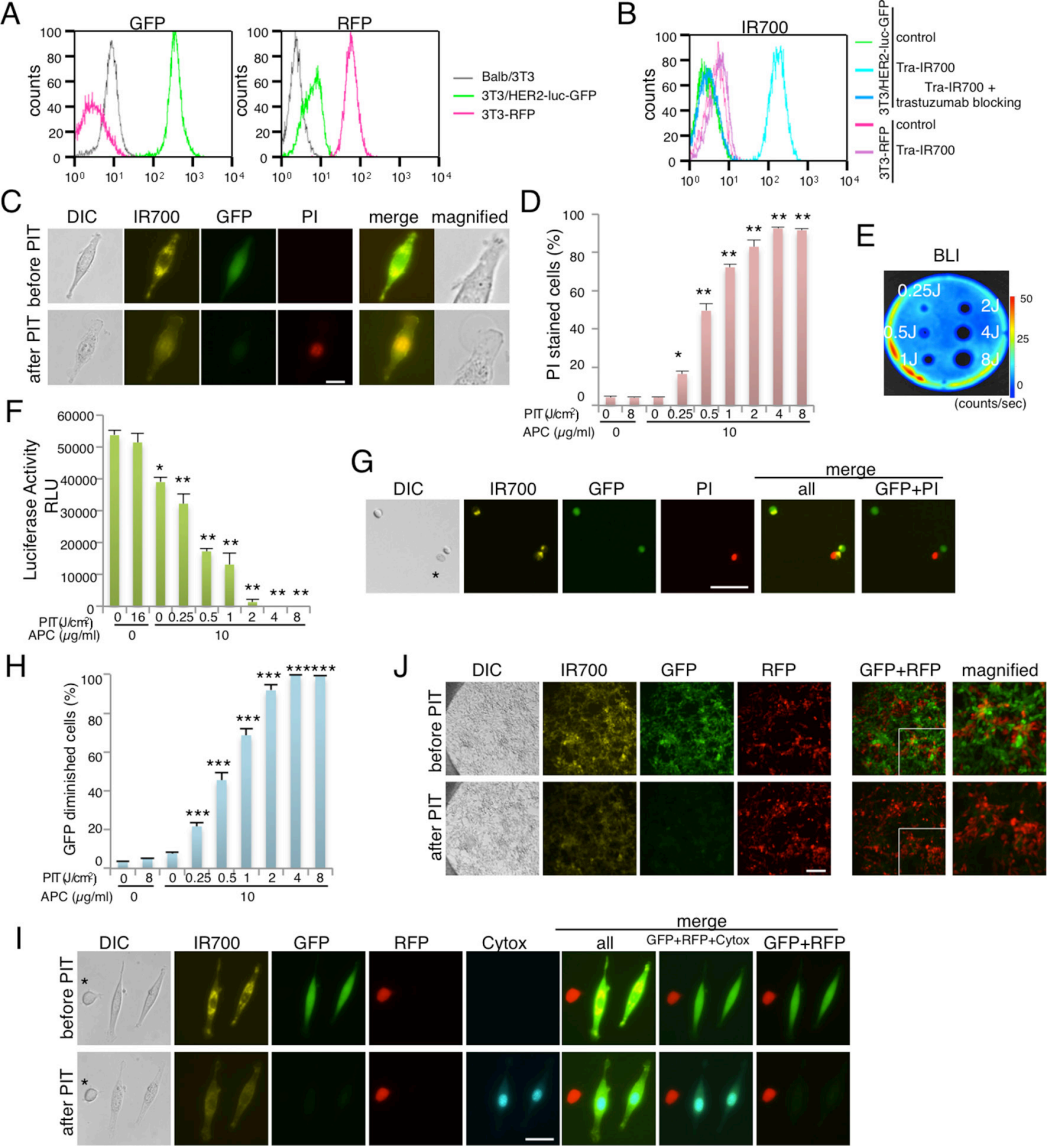
Effect of NIR-PIT on 2D cell monoculture and mixed cell culture **A.** Characterization of 3T3/HER2-luc-GFP cells and 3T3-RFP cells. **B.** 3T3/HER2-luc-GFP cells demonstrate HER2 expression. Specific binding was demonstrated in a blocking study with excess trastuzumab. Non-HER2 expressing Balb/3T3-RFP cells were also examined with tra-IR700 incubation, which confirmed no binding. **C.** 3T3/HER2-luc-GFP cells were incubated with tra-IR700 for 6 hr and observed before and after irradiation with NIR-light (2 J/cm^2^). Necrotic cell death was observed after exposure to NIR-light (1 hr after NIR-PIT). PI staining indicates necrotic cell death. Bar = 50 μm. **D.** Membrane damage and necrosis induced by NIR-PIT were measured by dead cell count using PI staining. Cell killing increased in a NIR-light dose-dependent manner (*n* = 4, *p < 0.001, **p < 0.0001, vs. untreated control, Student's *t* test). **E.** Bioluminescence imaging (BLI) of a treated 10 cm cell culture dish demonstrated that luciferase activity in 3T3/HER2-luc-GFP cells decreased in a NIR-light dose-dependent manner. **F.** Luciferase activity in 3T3/HER2-luc-GFP cells was measured as relative light unit (RLU), which also decreased in a NIR-light dose-dependent manner (*n* = 4, *p < 0.001, **p < 0.0001, vs. untreated control, Student's *t* test). **G.** 3T3/HER2-luc-GFP cells were incubated with tra-IR700 for 6 hr and irradiated with NIR-light (0.25 J/cm^2^). GFP-fluorescence intensity decreased in dead cells (*) stained by PI but was unchanged in living cells at 1 hr after NIR-PIT. Bar = 200 μm. **H.** GFP fluorescence intensity decreased after NIR-PIT in a NIR-light dose-dependent manner (*n* = 4, ***p < 0.0001, vs. untreated control, Student's *t* test). **I.** 3T3/HER2-luc-GFP cells were co-cultured with 3T3-RFP (non-HER2 expressing) cells. The mixture was treated with tra-IR700 and observed (before and after irradiation of NIR-light). Targeted specific necrotic cell death was observed upon excitation with NIR-light (2 J/cm^2^) 30 min after exposure; 3T3/HER2-luc-GFP cells were stained with dead staining Cytox Blue. No damage was demonstrated in the 3T3-RFP cells. *3T3-RFP cell, Bar = 25 μm. **J.** An almost confluent mix of 3T3/HER2-luc-GFP and 3T3-RFP cells was incubated with tra-IR700 for 6 hr and observed before and after irradiation with NIR-light (2 J/cm^2^). Bar = 200 μm.

To monitor the effect of NIR-PIT, the Balb/3T3 cell line was modified to express HER2, GFP and luciferase (3T3/HER2-luc-GFP). As a non-target of NIR-PIT, the Balb/3T3 cell line was optically modified to express RFP (3T3-RFP) (Figure 1A). The antibody-photosensitizer conjugate, trastuzumab-IR700 (tra-IR700), was incubated with 3T3/HER2-luc-GFP or 3T3-RFP cells, and fluorescence signals were evaluated by flow cytometry. After a 6-hr tra-IR700 incubation, 3T3/HER2-luc-GFP cells showed high fluorescence and were blocked by excess trastuzumab, which suggested specific binding. Almost no binding of tra-IR700 was observed in 3T3-RFP cells (Figure 1B). Serial fluorescence microscopy of 3T3/HER2-luc-GFP cells was performed before and after NIR-PIT with 690 nm light. After exposure to 2 J/cm^2^ NIR-light, cellular swelling and bleb formation were observed (Figure 1C). Time-lapse image analysis showed acute morphologic changes in the cell membrane, and cells incubated with propidium iodide (PI) demonstrated fluorescence of PI, which indicated cell death (Supplemental videos 1, 2). Most of these cellular changes were observed within 25 min of light exposure, which indicated the rapid induction of necrotic cell death after NIR-PIT. To quantify the effect of *in vitro* NIR-PIT, a cytotoxicity assay consisting of PI staining and luciferase activity was performed 1 hr after NIR-light irradiation. Based on the incorporation of PI, the cell death percentage increased in a light dose-dependent manner. No significant cytotoxicity was observed with either NIR light exposure or APC alone (Figure 1D). Bioluminescence showed significant decreases in relative light units (RLU) in NIR-PIT treated cells but not in controls (Figure 1E, 1F). BLI also showed a decrease in luciferase activity in a light dose-dependent manner (Figure 1F). GFP fluorescence intensity was greatly reduced in dead cells (stained positive with PI), whereas GFP fluorescence was preserved in surviving cells (Figure 1G). GFP fluorescence was reduced after NIR-PIT because the GFP was extruded from the cytoplasm after membrane rupture and was therefore markedly diluted and/or denatured. The GFP diminished cells increased in a light dose-dependent manner, and no significant change was detected with NIR-light exposure or APC alone based on FACS analysis (Figure 1H). Taken together, these data suggest that the effects of NIR-PIT on 3T3/HER2-luc-GFP could be monitored with both GFP fluorescence and bioluminescence *in vivo*. Next, to demonstrate selective elimination of target-expressing cells, a 2D mixed cell culture consisting of 3T3/HER2-luc-GFP and 3T3-RFP was used. Selective cell killing of 3T3/HER2-luc-GFP was documented with Cytox dead cell staining, and no significant changes were observed in HER2-negative 3T3-RFP cells after exposure to NIR-light, which suggested that NIR-PIT induced no damage in non-target cells that were immediately adjacent to target cells (Figure 1I). Elimination of 3T3/HER2-luc-GFP cells from an almost-confluent 2D mixed cell culture was demonstrated after NIR-PIT (Figure 1J).

### Characterization of *in vitro* 3D culture and target specific cell killing with NIR-PIT in a mixed 3D culture

**Figure 2:**
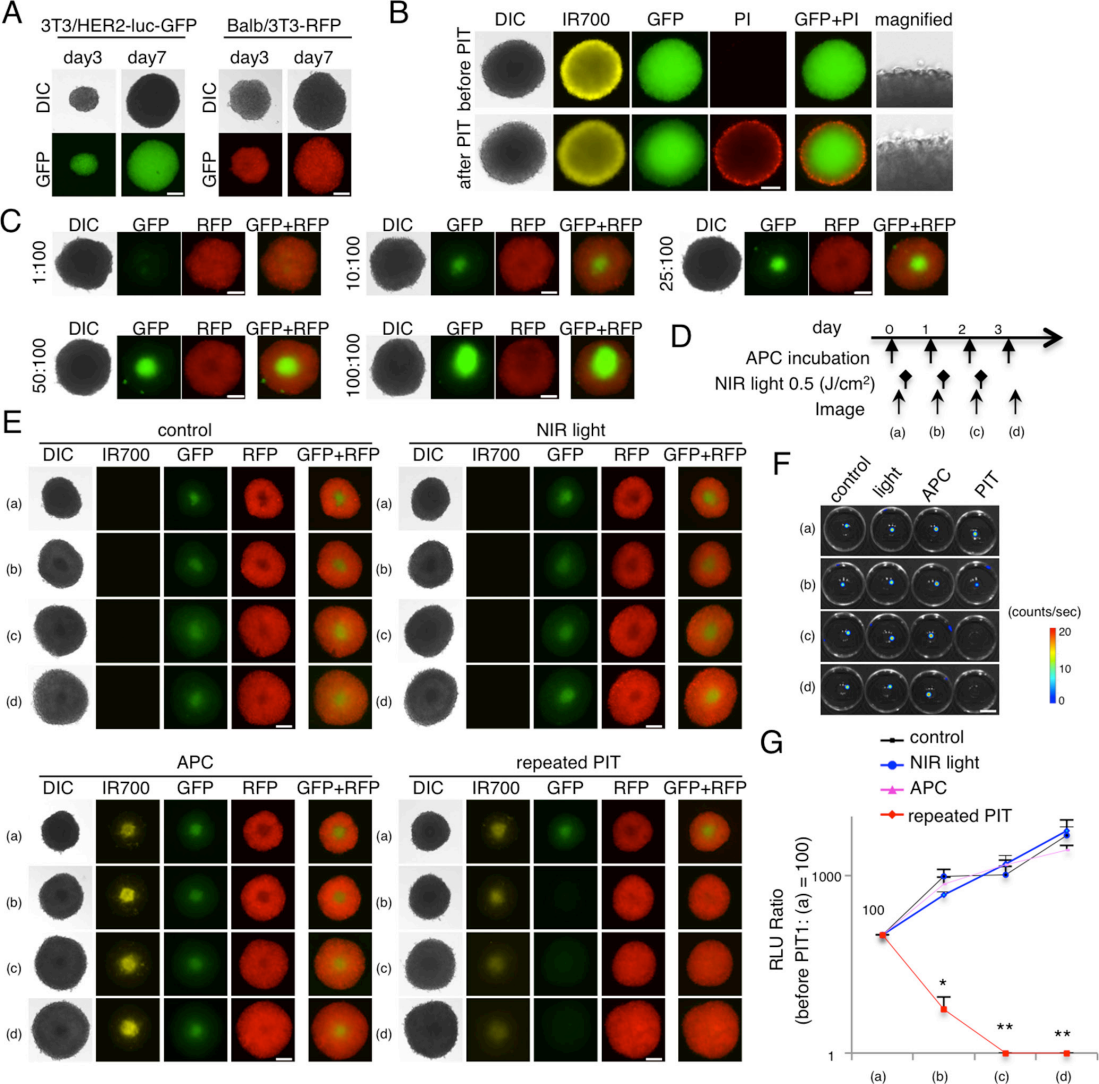
Evaluation of NIR-PIT effect on 3D cell monoculture and 3D mixed cell culture **A.** Representative image of 3T3/HER2-luc-GFP and 3T3-RFP 3D spheroids at day 3 and day 7. Bar = 200 μm. **B.** 3D spheroid at day 7 after 6 hr incubation with tra-IR700, before and after irradiation of NIR-light (0.5 J/cm^2^). Necrotic cell death was observed in the layer exhibiting IR700 fluorescence 1 hr after NIR-light. Bar = 200 μm. **C.** Representative images of various ratios of mixed spheroid at day 7. Bar = 200 μm. **D.** The NIR-PIT regimen incorporating repeated NIR-light exposures is shown. **E.** Day 7 3T3/HER2-luc-GFP and 3T3-RFP mixed 3D spheroids were divided into 4 groups as shown. GFP fluorescence was greatly diminished after the 2nd NIR-PIT, whereas other cell types grew. Bar = 200 μm. **F.** BLI of each group demonstrated that luciferase activity decreased after repeated NIR-PIT. Bar = 10 mm. **G.** Luciferase activity in mixed 3D spheroids gradually decreased after repeated NIR-PIT, leading to complete killing of cells in the spheroid (*n* = 5) (*p = 0.0092 < 0.01, **p < 0.0001 vs control, Tukey's test with ANOVA).

Next, we evaluated the efficacy of NIR-PIT in 3D spheroids (Figure 2). Fluorescence images of 3T3/HER2-luc-GFP or 3T3-RFP spheroids at day 3 or day 7 were obtained (Figure 2A). NIR-PIT caused necrotic cell death in the APC-bound outer layer of the spheroid (Figure 2B). After exposure to NIR-light, the spheroid immediately expanded, and the outer cells became necrotic, as demonstrated by staining with PI (Supplemental videos 3, 4). These changes occurred within 25 min of light exposure. To demonstrate target specific cell killing in the 3D cell culture, a mixed 3D spheroid that consisted of the two described cell lines was established (Figure 2C). Daily repeated NIR-PIT (Figure 2D) achieved complete eradication of the 3T3/HER2-luc-GFP cell population (diminishing GFP fluorescence) in the mixed 3D spheroid, and 3T3-RFP cells and other controls continued to grow (Figure 2E), as confirmed by BLI (*p = 0.0092 < 0.01, **p < 0.0001 vs control, Tukey's test with ANOVA) (Figure 2F, 2G). These results suggest that repeated NIR-PIT could eradicate target-expressing cells (3T3/HER2-luc-GFP) that were growing in mixed 3D spheroids without damaging non-target cells (3T3-RFP). Unlike 2D cultures in which the APC could infiltrate the entire cell mixture and in which one NIR exposure was sufficient, 3D cultures required repeated NIR exposure because the APC entered new parts of the spheroid after every treatment.

### NIR-PIT prevents formation of metastatic disease *in vivo*

**Figure 3:**
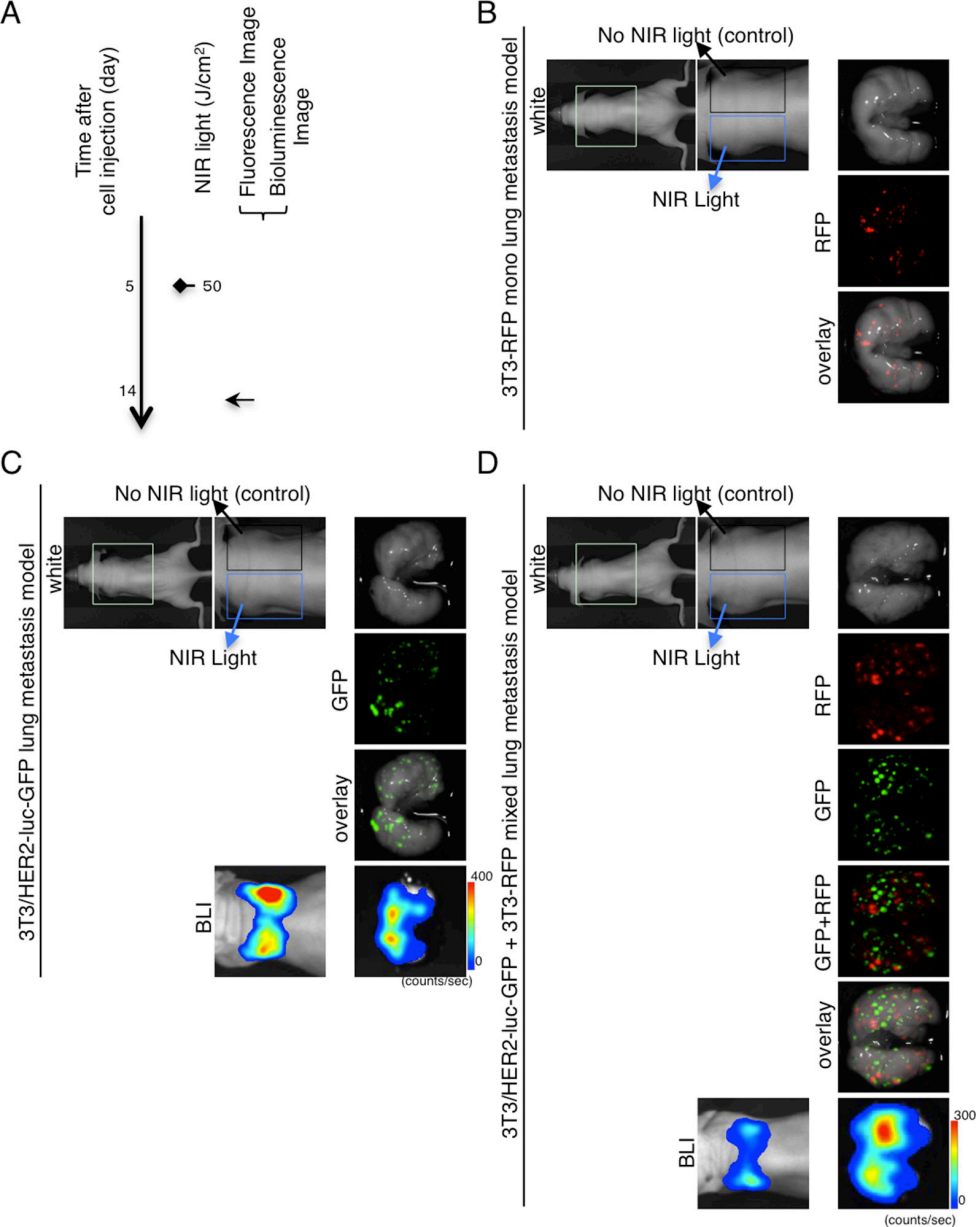
Demonstration of NIR-irradiation effects without intravenous APC injection in lung metastasis model **A.** The regimen of NIR-irradiation without intravenous APC injection is shown. Images were obtained at each time point as indicated. NIR-irradiation was administered to the left lung. **B.** *Ex vivo* fluorescence imaging (RFP) of 3T3-RFP lung metastasis model. **C.** *In vivo* bioluminescence and *ex vivo* fluorescence/bioluminescence imaging of 3T3/HER2-luc-GFP lung metastasis model. **D.** *In vivo* bioluminescence and *ex vivo* fluorescence/bioluminescence imaging of 3T3/HER2-luc-GFP (target) and 3T3-RFP (non-target) mixed lung metastasis model. **B–D.** No remarkable preventive effect was detected between the left and right lung. The appearance of both left and right lung was intact.

*In vivo* mouse models of lung metastases consisting of 3T3-RFP, 3T3/HER2-luc-GFP, and mixtures of 3T3-RFP and 3T3/HER2-luc-GFP were established and monitored by fluorescence imaging and BLI (Figure 3A). The treatment and imaging regimen is shown (Figure 3A). NIR-irradiation was administered only to the left lung such that the relative effects could be observed in each mouse. The NIR-irradiation effect was confirmed with GFP/RFP-fluorescence or bioluminescence, and there was no obvious difference between the left NIR-irradiation lung and the right lung in each model (Figure 3B, 3C, 3D).

**Figure 4:**
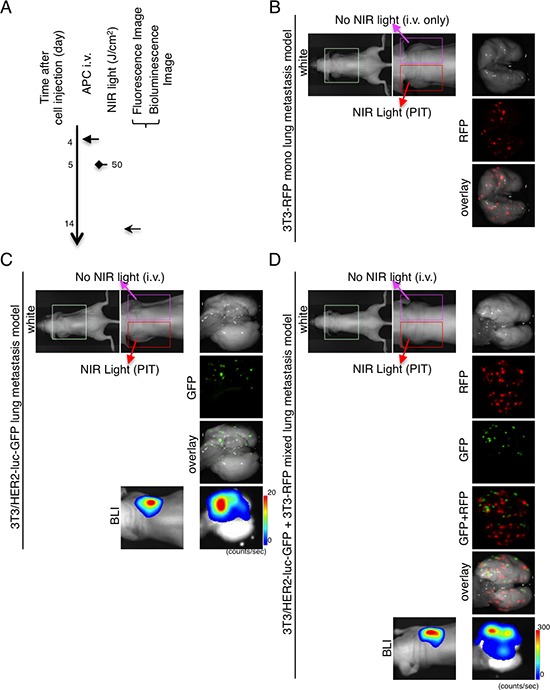
Demonstration of treatment effects of NIR-PIT in lung metastasis model receiving both APC and NIR-irradiation **A.** The regimen of NIR-PIT is shown. Images were obtained at each time point as indicated. NIR-PIT was administered to the left lung in the same manner as Figure 3. **B.** *Ex vivo* fluorescence imaging (RFP) of 3T3-RFP lung metastasis model shows no preventive effect. **C.** *In vivo* bioluminescence and *ex vivo* fluorescence/bioluminescence imaging of 3T3/HER2-luc-GFP lung metastasis model shows distinct decreases of GFP-fluorescence and bioluminescence in the left lung. **D.** *In vivo* bioluminescence and *ex vivo* fluorescence/bioluminescence imaging of 3T3/HER2-luc-GFP (target) and 3T3-RFP (non-target) mixed lung metastasis model. Remarkable decreases in GFP-fluorescence and bioluminescence in the left lung were observed, but there was no change in RFP-fluorescence between the left and right lung. **B–D.** The appearance of both left and right lung was intact.

Next, to investigate whether NIR-PIT could specifically prevent metastases, NIR-PIT was administered to the left lung of the mouse models of lung metastases and consisted of 3T3-RFP, 3T3/HER2-luc-GFP, and mixtures of 3T3-RFP and 3T3/HER2-luc-GFP (Figure 4A). NIR-PIT was administered only to the left lung such that the relative effects could be observed in each mouse. NIR-PIT diminished the IR700-fluorescence on the left side of the chest (Supplemental Figure 1). The NIR-PIT caused decreases in both bioluminescence and GFP-fluorescence in the left lung of the 3T3/HER2-luc-GFP and 3T3-RFP mixed mouse model, but no effect on RFP-fluorescence was detected in the 3T3-RFP and 3T3-RFP+3T3/HER2-luc-GFP mixed lung metastasis mouse model (Figure 4B, 4C, 4D). Moreover, the appearance of the lung gave no indication that NIR-PIT caused damage to the treated left lung. Collectively, these data suggest that NIR-PIT can prevent target-specific metastasis without inducing harm to the normal lung tissue or affecting non-target metastasis formation.

### Quantification of the effect of NIR-PIT on lung metastasis model

**Figure 5:**
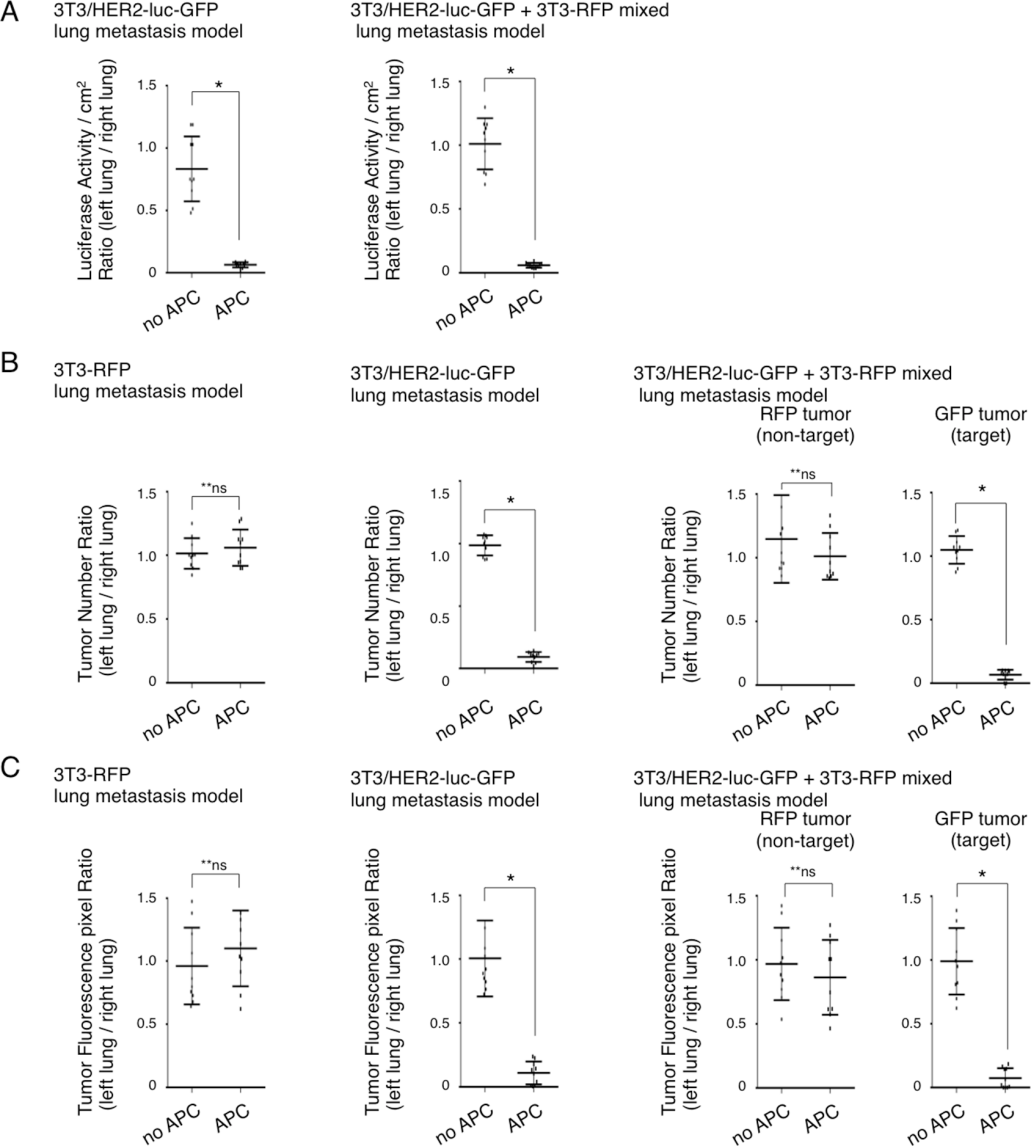
Quantification of NIR-PIT effects on lung metastasis model **A.** Quantification of luciferase activity on *ex vivo* 3T3/HER2-luc-GFP and 3T3/HER2-luc-GFP and 3T3-RFP mixed metastasis model. Luciferase activity/cm^2^ between left and right lung was significantly decreased in the left lung with NIR-PIT (n = 10 in each group) (*p < 0.0001, Mann-Whitney *U*-test). **B.** Quantification of the number of metastases with fluorescence imaging on *ex vivo* specimens of the 3T3-RFP, 3T3/HER2-luc-GFP, and 3T3/HER2-luc-GFP +3T3-RFP mixed metastasis models. The number of GFP positive tumors was significantly decreased in the left lung after NIR-PIT, whereas the number of RFP positive tumors was unchanged (*n* = 10 in each group) (*p < 0.0001, **n.s., Mann-Whitney *U*-test). **C.** Quantification of fluorescence pixels on *ex vivo* 3T3-RFP, 3T3/HER2-luc-GFP, and 3T3/HER2-luc-GFP and 3T3-RFP mixed metastasis model. GFP fluorescence was significantly decreased in the left lung after NIR-PIT, whereas RFP fluorescence pixels were unchanged (*n* = 10 in each group) (*p < 0.0001, **n.s., Mann-Whitney *U*-test).

To quantify the metastasis prevention effect with NIR-PIT, the luciferase activity, number of metastatic tumor nodules, and GFP/RFP fluorescence between the right and left lung were evaluated in NIR-irradiated and NIR-PIT mice (Figures 3, 4). Luciferase activity was reduced after NIR-PIT in mice with 3T3/HER2-luc-GFP and in the 3T3-RFP+3T3/HER2-luc-GFP mixed lung metastasis mouse model (*p < 0.0001, Mann-Whitney *U*-test) (Figure 5A). Numeric evaluation revealed that NIR-PIT significantly reduced target GFP tumors, but no significant change was observed in the RFP tumors (*p < 0.0001, **n.s., Mann-Whitney *U*-test) (Figure 5B). Finally, fluorescence pixel ratios also showed that NIR-PIT significantly reduced the target GFP-tumor fluorescence pixels compared with non-target RFP-tumor fluorescence pixels (*p < 0.0001, **n.s., Mann-Whitney *U*-test) (Figure 5C). Collectively, these results confirmed that NIR-PIT significantly and selectively reduced lung metastases in this mouse model without damaging the adjacent normal lung tissue or non-target tumors (Figures 3, 4).

## DISCUSSION

In this study, NIR-PIT significantly and selectively prevented the growth of early lung metastasis in a mouse model. To non-invasively evaluate therapeutic effects *in vivo*, we used fluorescence and bioluminescence reporters because each modality provided complimentary information. Multimodal imaging has been shown to demonstrate a more nuanced understanding of the therapeutic effects in mice [8–10]. BLI, using firefly luciferase, was used as the primary quantitative outcome measure in this study. This is a well-established method of determining *in vivo* viability [11, 12] because the BLI reaction requires both oxygen and ATP to actively transport the substrate luciferin and subsequently catalyze the photochemical reaction [12]. Because NIR-PIT induced necrotic cell death, which releases cellular ATP, BLI is a good biomarker for the functional effects of NIR-PIT [6, 13]. Fluorescence imaging enables the full process of tumorigenesis, treatment, regression, metastasis, or recurrence [14, 15]. The high level of tumor fluorescence allowed us to perform imaging and quantification of tumor growth and dissemination without using additional injections of contrast agents or substrates. Interestingly, although visible inspection of the lung often indicated no abnormality, fluorescence imaging revealed many invisible early stage metastases [15]. This approach enabled the more precise quantification of the number and fluorescence of tumors in the *ex vivo* lung. By employing cytoplasmic GFP expressing cells, the antitumor effects induced by NIR-PIT could be clearly monitored due to the extrusion of GFP from treated cells [7]. RFP fluorescence was not significantly affected because it was expressed only in non-target cells, which enabled the confirmation of highly selective cell killing. Thus, with this combination of *in vivo* imaging, we demonstrated the significant and selective prevention of lung metastases.

We used a lung metastasis model that utilized tail vein cell injection [16]. Surgical orthotopic injection (SOI) is another method of creating a lung metastasis model [17]. Various advantages and disadvantages exist between these models. SOI requires highly trained skills and invasive methods and results in high pre-procedure mortality rate, but it is thought to be a more physiological model. The cell-injection model has the advantages of less advanced skills, less invasiveness to animals, and a very low pre-procedure mortality rate. Due to these considerations and the recent requirement of animal wellness, we used cell-injection models. Moreover, in a mice lung metastasis model, it is difficult to observe live fluorescence imaging because the mice die due to pneumothorax. To prevent this pneumothorax, a ventilation system for mice was necessary, and it demanded advanced skills, invasive procedures, and expensive devices [18]. Thus, we monitored live cell-injection lung metastasis models by non-invasive BLI and then confirmed our findings with *ex vivo* fluorescence imaging.

The metastatic process consists of several steps: 1. loss of cellular adhesion, increased motility and invasiveness, which leads to 2. intravasation into the circulatory system, 3. transport by circulation, 4. adherence to distant organ vessel walls, 5. extravasation, and 6. colonization at a distant site [19–22]. Despite recent advances in the understanding of this process, metastases remain the major cause of cancer-associated mortality. Unfortunately, systemic therapies that are administered pre-emptively are typically unsuccessful in suppressing metastases [19]. In this study, by targeting metastatic cancer cells at steps 4, 5, and 6 described above, NIR-PIT was able to prevent the establishment and/or growth of metastases in the lung. NIR-PIT can treat cancer cells that have access to the APC and NIR-light. Thus, in the vessel, where tumor cells are at the time of extravasation and at colonization, they are susceptible to NIR-PIT, particularly in the lung, where the transmission of light is optimal.

In addition to metastases, NIR-PIT could be used at the primary site, especially during or after surgery to “mop up” residual cancer cells. This therapy could also treat circulating tumor cells (CTCs), at least within the superficial vessels such as arteries and veins in the skin. The ability to prevent the growth of metastases is another possible role for NIR-PIT.

Some malignancies, such as sarcomas, preferentially metastasize to the lungs [23]. This factor has prompted the use of surgical removal of oligometastases as a strategy to prolong survival. It is possible that NIR-PIT could be particularly useful in these tumor types as a method for preventing pulmonary metastases. Naturally, this approach would require the identification of an antibody reliably associated with sarcomas [24]. Unlike the mouse, in which NIR-light can be administered transcutaneously, in the human, NIR-light would likely be administered bronchoscopically or thoracoscopically. Compared with surgery, NIR-PIT has the distinct advantage of minimal invasiveness and not incurring damage to the normal lung parenchyma. It could also be repeated multiple times in the same patient if suspicions of additional pulmonary metastases arise. This process could enable the preservation of pulmonary reserve in addition to reducing, if not eliminating, the impact of metastatic disease. Of course, NIR-PIT would have less impact if there were other sites of metastases that were less amenable to NIR-PIT.

There are several limitations to this study. First, few pulmonary metastases overexpress HER2, which was simply used as an exemplar in this study. However, NIR-PIT has proven to be effective with multiple antibodies, including anti-EGFR, anti-PSMA, anti-mesothelin, and anti-CEA [5–7, 13, 25, 26]. Therefore, we are optimistic that NIR-PIT could be used with a wide range of antibodies and cancers. The possibility of mixtures of APCs is also feasible in cases in which there are mixed populations of antigen expressing cells [27]. Due to limited light penetration, translation to humans would require bronchoscopy or direct thoracoscopy with fibrooptics to expose light to the entire lung. Metastases outside the lungs would not be affected by NIR-PIT, and therefore, the procedure may have little impact in widespread metastatic disease. However, because the lungs are often the most symptomatic, NIR-PIT may provide palliative relief and modest survival benefits in the presence of extrapulmonary metastases. Another limitation is that the long-term durability of response and side effects of NIR-PIT were not determined. However, short-term studies of the mice demonstrated only temporary minimal body weight loss after NIR-PIT.

In conclusion, this study demonstrates that NIR-PIT can be used as a local treatment of early pulmonary metastases and can therefore be viewed as a preventive therapy for pulmonary metastases.

## MATERIALS AND METHODS

### Reagents

Water soluble, silicon-phthalocyanine derivative, IRDye 700DX NHS ester was obtained from LI-COR Bioscience (Lincoln, NE, USA). Trastuzumab, 95% humanized IgG_1_ mAb directed against HER2, was purchased from Genentech (South San Francisco, CA, USA). All other chemicals were of reagent grade.

### Synthesis of IR700-conjugated trastuzumab

Conjugation of dyes with mAbs was performed according to previous reports [13]. Briefly, trastuzumab (1 mg, 6.8 nmol) was incubated with IR700 NHS ester (60.2 μg, 30.8 nmol) in 0.1 mol/L Na_2_HPO_4_ (pH 8.6) at room temperature for 1 hr. The mixture was purified with a Sephadex G25 column (PD-10; GE Healthcare, Piscataway, NJ, USA). The protein concentration was determined with the Coomassie Plus protein assay kit (Thermo Fisher Scientific Inc, Rockford, IL, USA) by measuring absorption at 595 nm (8453 Value System; Agilent Technologies, Santa Clara, CA, USA). The concentration of IR700 was measured by absorption at 689 nm to confirm the number of fluorophore molecules conjugated to each mAb. The synthesis was controlled such that an average of three IR700 molecules were bound to a single antibody. We performed SDS-PAGE as a quality control for each conjugate as previously reported [5]. We abbreviate IR700 conjugated to trastuzumab as tra-IR700.

### Cell culture

HER2 and luciferase/GFP-expressing NIH3T3 cells (3T3/HER2-luc-GFP) were established with a transfection of RediFect Red-FLuc-GFP (PerkinElmer, Waltham, MA, USA). High GFP and luciferase expression was confirmed with 10 passages. Balb/3T3 cells stably expressing RFP were established with transfection by RFP (EF1a)-Neo lentiviral particles (AMSBIO, Cambridge, MA, USA). High RFP expression was confirmed in the absence of a selection agent with 10 passages. 3T3 cells stably expressing RFP (3T3-RFP) were used as negative controls [13]. Cells were grown in RPMI 1640 (Life Technologies, Gaithersburg, MD, USA) supplemented with 10% fetal bovine serum and 1% penicillin/streptomycin (Life Technologies) in tissue culture flasks in a humidified incubator at 37°C at an atmosphere of 95% air and 5% carbon dioxide.

### 3D spheroid culture

Spheroids were generated by the hanging drop method in which five thousand cells were suspended in 50 μL medium and were then dispensed into 96 well plates (3D Biomatrix Inc, Ann Arbor, MI, USA) following the manufacturer's instructions [13]. Mixed spheroids were made with 5,000 cells of Balb/3T3-RFP and 500 cells of 3T3/HER2-luc-GFP (100:10). After observation or treatment, spheroids were again incubated with the hanging drop plates containing new media.

### Flow cytometry

Fluorescence from cells incubated with tra-IR700, GFP fluorescence or RFP fluorescence was measured using a flow cytometer (FACS Calibur, BD BioSciences, San Jose, CA, USA) and analyzed with CellQuest software (BD BioSciences). 3T3/HER2-luc-GFP or 3T3-RFP cells (1 × 10^5^) were incubated with tra-IR700 for 6 hr at 37°C. To validate the specific binding of the conjugated antibody, excess antibody (50 μg) was used to block 0.5 μg of APCs [6].

### Fluorescence microscopy

To detect the antigen specific localization of IR700 conjugates, fluorescence microscopy was performed (IX81; Olympus America, Melville, NY, USA). Ten thousand cells were seeded on cover-glass-bottomed dishes and incubated for 24 hr. Tra-IR700 was then added to the culture medium at 10 μg/mL and incubated at 37°C for 6 hr. The cells were then washed with PBS; Propidium Iodide (PI) (1:2000) (Life Technologies) and Cytox Blue (1:500) (Life Technologies) were used to detect dead cells. They were added into the media 30 min before observation. The cells were then exposed to NIR-light (2 J/cm^2^), and serial images were obtained. The filter was set to detect IR700 fluorescence using a 590–650 nm excitation filter and a 665–740 nm band pass emission filter.

The images were analyzed using the ImageJ software (http://rsb.info.nih.gov/ij/) [28].

### *In vitro* NIR-PIT

One hundred thousand cells were seeded into 24-well plates, or ten million cells were seeded into a 10 cm dish and incubated for 24 hr. The medium was replaced with fresh culture medium containing 10 μg/mL of tra-IR700 that was incubated for 6 hr at 37°C. After washing with PBS, phenol red free culture medium was added. Then, cells were irradiated with a NIR laser, which emits light at a 685 to 695 nm wavelength (BWF5-690-8-600-0.37; B&W TEK INC., Newark, DE, USA). The output power density in mW/cm^2^ was measured with an optical power meter (PM 100, Thorlabs, Newton, NJ, USA).

### Cytotoxicity/Phototoxicity assay

The cytotoxic effects of NIR-PIT with tra-IR700 were determined by luciferase activity and flow cytometry of PI staining. For luciferase activity, 150 μg/mL of D-luciferin-containing media (Gold Biotechnology, St Louis, MO, USA) was administered to PBS-washed cells 1 hr after NIR-PIT, which were analyzed on a bioluminescence imaging (BLI) system (Photon Imager; Biospace Lab, Paris, France). For the flow cytometry assay, cells were trypsinized 1 hr after treatment and washed with PBS. PI was added to the cell suspension (final 2 μg/mL), and the cells were incubated at room temperature for 30 min prior to flow cytometry. Residual GFP fluorescence at 1 hr after NIR-PIT was also evaluated with FACS [7].

### Animal and tumor models

All *in vivo* procedures were conducted in compliance with the Guide for the Care and Use of Laboratory Animal Resources (1996), US National Research Council, and approved by the local Animal Care and Use Committee. Six- to eight-week-old female homozygote athymic nude mice were purchased from Charles River (NCI-Frederick). During the procedures, mice were anesthetized with isoflurane.

To evaluate targeted metastasis prevention with NIR-PIT in the lung metastasis mouse model [16], two million 3T3/HER2-luc-GFP cells in PBS (total 200 μL) for mono-lung metastasis were intravenously injected through the tail vein. Two million 3T3-RFP cells in PBS (total 200 μL) for the 3T3-RFP lung metastasis mouse model and a mixture of one million 3T3/HER2-luc-GFP cells and one million 3T3-RFP cells in PBS (total 200 μL) for the mixed lung metastasis mouse model were intravenously injected through the tail vein.

### *In vivo* fluorescence imaging

*In vivo* fluorescence images were obtained using a Pearl Imager (LI-COR Bioscience) to detect IR700 fluorescence, and a Maestro Imager (CRi, Woburn, MA, USA) was used to detect GFP/RFP. A band-pass filter from 445 to 490 nm (excitation), and a long-pass blue filter over 515 nm (emission) was used for GFP analysis; 503 to 555 nm (excitation) and a long-pass green filter over 580 nm (emission) were used for RFP analysis. The tunable emission filter was automatically increased in 10-nm increments from 515 to 580 nm at a constant exposure time (800 msec). The spectral fluorescence images consisted of autofluorescence spectra and the spectra from GFP/RFP (tumor), which were then unmixed based on the characteristic spectral pattern of GFP using Maestro software (CRi). Regions of interest (ROIs) were manually drawn on either the left or right lung, as appropriate to the model, and the fluorescence intensity and pixels were measured [7]. The number of the tumor nodules in the left or right lung was calculated.

### *In vivo* bioluminescence imaging

For BLI, D-luciferin (15 mg/mL, 200 μL) was injected intraperitoneally, and the mice were analyzed with a Photon Imager for luciferase activity at day 14. To quantify the luciferase activities, ROIs of the entire left lung and entire right lung *in vivo/ex vivo* were obtained. To account for the differences in volume between the left and right lung, the signal was recorded as photon counts/cm^2^.

### *In vivo* NIR-PIT

Mice were randomized into 2 groups of 10 animals and divided into four groups based on local NIR-irradiation (in the left or right lung) for the following treatments (see Figure 3A, 4A): (1) right lung with no treatment (control); (2) left lung with local NIR-light exposure only at 50 J/cm^2^ on day 5 after cell injection; (3) right lung with 100 μg of tra-IR700 i.v. on day 4 but no NIR-light exposure on day 5 after cell injection; and (4) left lung with 100 μg of tra-IR700 i.v. on day 4 after cell injection followed by NIR-light at 50 J/cm^2^ one day after intravenous injection of tra-IR700 (treatment group). The mice were irradiated with NIR-light from 2 directions (each 25 J/cm^2^) via the left back and left chest. IR700 fluorescence imaging was performed before and after NIR-PIT. Serial fluorescence imaging (RFP/GFP) and BLI were obtained at 14 days after cell injection (see Figures 3A, 4A).

### Statistical analysis

Data are expressed as the means ± s.e.m. from a minimum of four experiments, unless otherwise indicated. Statistical analyses were performed using a statistics program (GraphPad Prism; GraphPad Software, La Jolla, CA, USA). For multiple comparisons, a one-way analysis of variance (ANOVA) with Tukey's test was used. Student's *t* test was used to compare the two *in vitro* 2D culture studies. To compare the tumor left/right ratio in the lung, a Mann-Whitney *U*-test was used. *p* < 0.05 was considered to indicate a statistically significant difference.

## SUPPLEMENTARY FIGURE AND VIDEOS










