# Supplementary material for: Near infrared photoimmunotherapy prevents lung cancer metastases in a murine model
Source: Oncotarget. 2015 May 13;6(23):19747–58. doi: 10.18632/oncotarget.3850 (PMC4637318; doi:10.18632/oncotarget.3850)
Supplement: Supplementary file 1 [file oncotarget-06-19747-s001.pdf]

SUPPLEMENTARY FIGURE AND VIDEOS

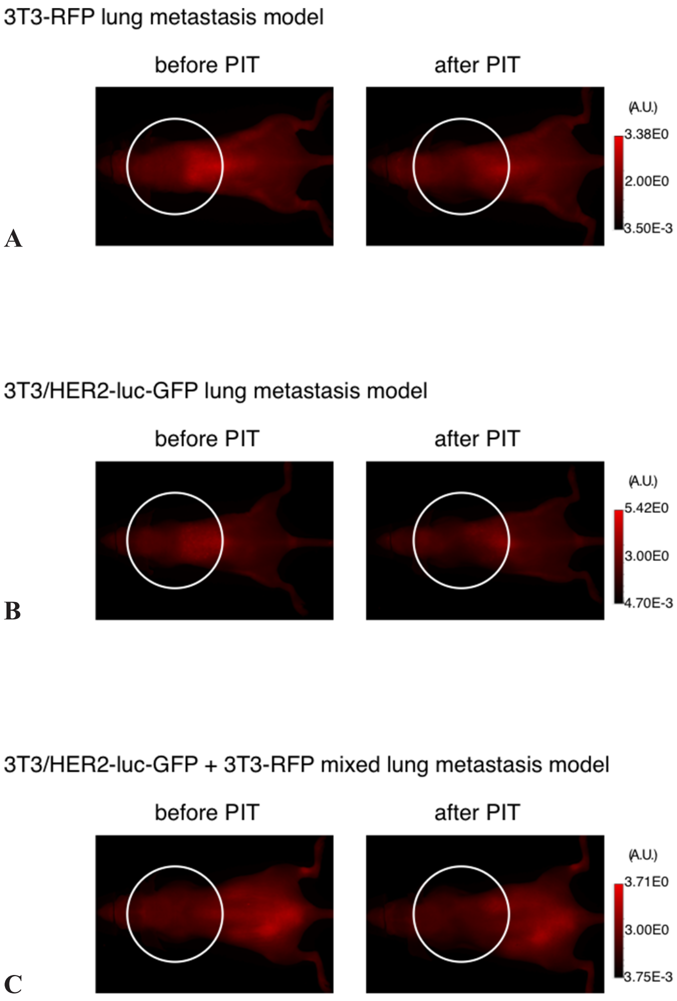

Supplementary Figure S1: *In vivo* IR700 fluorescence imaging in response to NIR-PIT. *In vivo* IR700 fluorescence imaging in response to NIR-PIT. IR700 fluorescence was decreased on the left side of the back in response to NIR-PIT **A.** 3T3-RFP lung metastasis model, **B**. 3T3/HER2-luc-GFP lung metastasis model, **C**. 3T3-RFP and 3T3/HER2-luc-GFP mixed lung metastasis model by NIR-PIT).

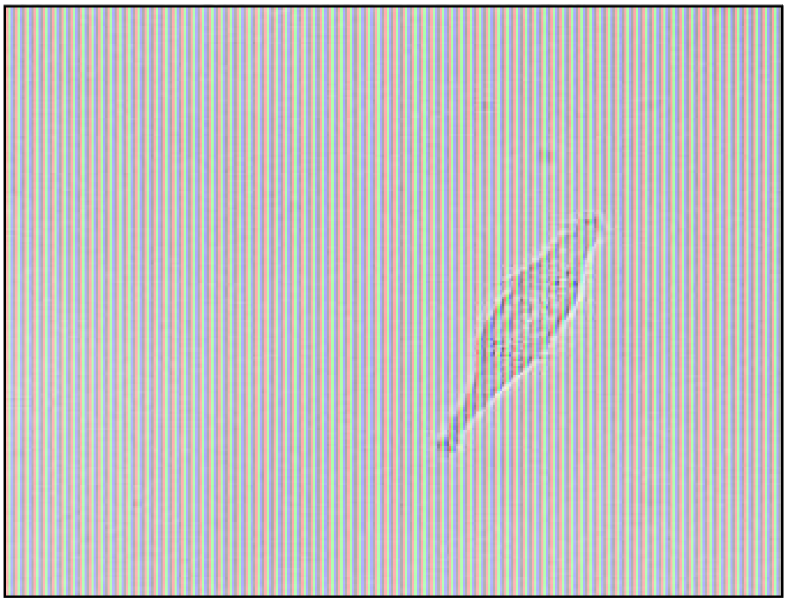

Supplementary Video S1: Time -lapse Imaging of NIR-PIT *in vitro* 2D culture. Time-lapse sequential images shows morphologic changes of the cell and rapid membrane damage detected by PI staining after NIR-light irradiation in cells treated with tra-IR700 (for 25 min observation in total). Flashing light is NIR-light irradiation (2 J/cm^2).
video S1: DIC time-lapse image of 3T3/HER2-luc-GFP cell treated by NIR-PIT.

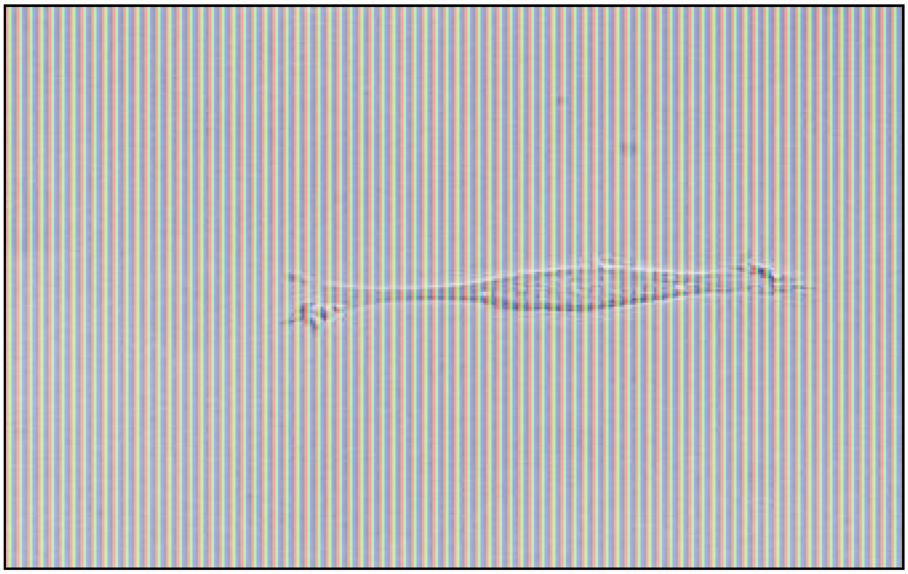

Supplementary Video S2: Time -lapse Imaging of NIR-PIT *in vitro* 2D culture. Time-lapse sequential images shows morphologic changes of the cell and rapid membrane damage detected by PI staining after NIR-light irradiation in cells treated with tra-IR700 (for 25 min observation in total). Flashing light is NIR-light irradiation (2 J/cm^2).
video S2:Fluorescence of PI time-lapse image of 3T3/HER2-luc-GFP cell treated by NIR-PIT.

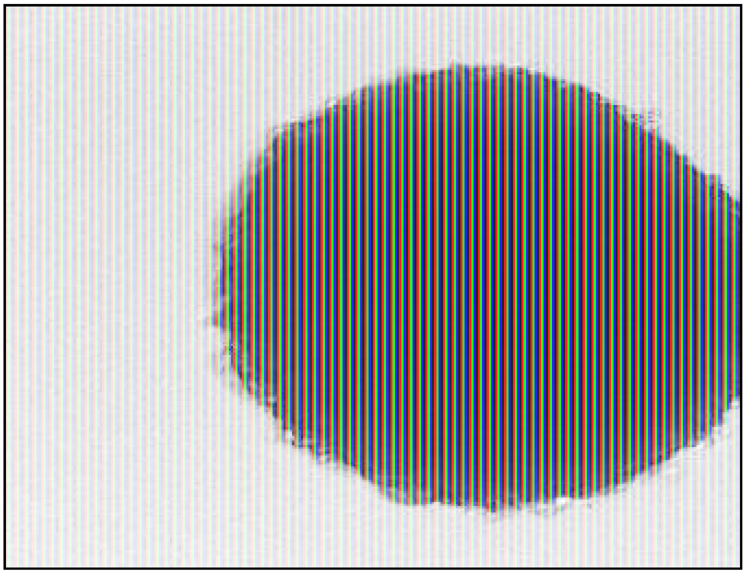

Supplementary Video S3: Time-lapse Imaging of NIR-PIT *in vitro* 3D culture. Time-lapse sequential images showed ballooning of the spheroid and rapid membrane damage detected by PI staining after NIR-light irradiation in a spheroid treated with tra-IR700 (5 sec intervals, total 25 min observation). Flashing light is NIR-light irradiation (2 J/cm2).
video S3: DIC time-lapse image of 3T3/HER2-luc-GFP spheroid treated by NIR-PIT.

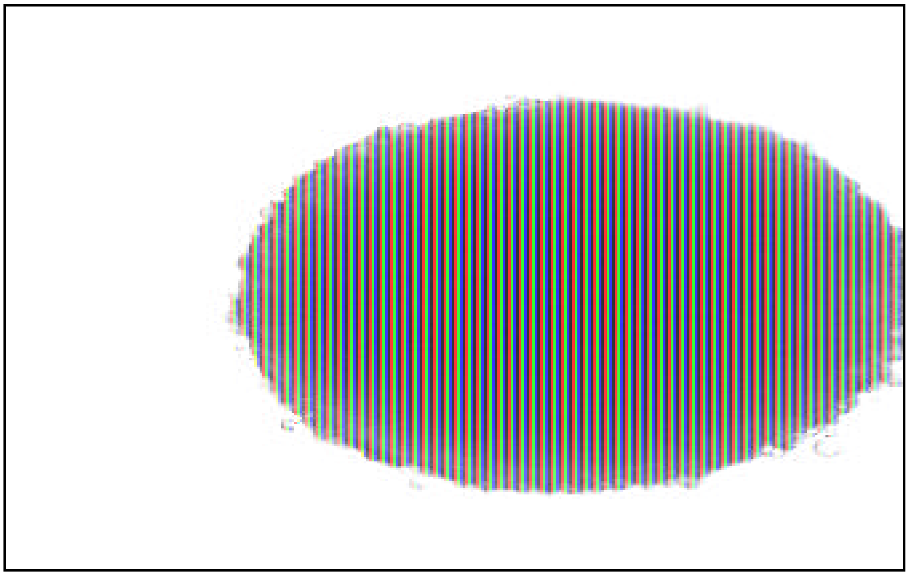

Supplementary Video S4: Time-lapse Imaging of NIR-PIT *in vitro* 3D culture. Time-lapse sequential images showed ballooning of the spheroid and rapid membrane damage detected by PI staining after NIR-light irradiation in a spheroid treated with tra-IR700 (5 sec intervals, total 25 min observation). Flashing light is NIR-light irradiation (2 J/cm2).

video S4: Fluorescence of PI time-lapse image of 3T3/HER2-luc-GFP spheroid treated by NIR-PIT.
